# MicroRNAs: From Female Fertility, Germ Cells, and Stem Cells to Cancer in Humans

**DOI:** 10.1155/2016/3984937

**Published:** 2015-11-09

**Authors:** Irma Virant-Klun, Anders Ståhlberg, Mikael Kubista, Thomas Skutella

**Affiliations:** ^1^Department of Obstetrics and Gynaecology, University Medical Center Ljubljana, SL-1000 Ljubljana, Slovenia; ^2^Sahlgrenska Cancer Center, Department of Pathology, Institute of Biomedicine, University of Gothenburg, 40530 Gothenburg, Sweden; ^3^TATAA Biocenter, 41103 Gothenburg, Sweden; ^4^Institute of Biotechnology, Czech Academy of Sciences, 14220 Prague, Czech Republic; ^5^Institute for Anatomy and Cell Biology, University of Heidelberg, 69120 Heidelberg, Germany

## Abstract

MicroRNAs are a family of naturally occurring small noncoding RNA molecules that play an important regulatory role in gene expression. They are suggested to regulate a large proportion of protein encoding genes by mediating the translational suppression and posttranscriptional control of gene expression. Recent findings show that microRNAs are emerging as important regulators of cellular differentiation and dedifferentiation, and are deeply involved in developmental processes including human preimplantation development. They keep a balance between pluripotency and differentiation in the embryo and embryonic stem cells. Moreover, it became evident that dysregulation of microRNA expression may play a fundamental role in progression and dissemination of different cancers including ovarian cancer. The interest is still increased by the discovery of exosomes, that is, cell-derived vesicles, which can carry different proteins but also microRNAs between different cells and are involved in cell-to-cell communication. MicroRNAs, together with exosomes, have a great potential to be used for prognosis, therapy, and biomarkers of different diseases including infertility. The aim of this review paper is to summarize the existent knowledge on microRNAs related to female fertility and cancer: from primordial germ cells and ovarian function, germinal stem cells, oocytes, and embryos to embryonic stem cells.

## 1. Introduction

It is estimated that only approximately 2% of the human genome represents the protein-coding region. More and more, it turns out that the key factor of this phenomenon may be microRNAs (miRNAs, miRs). It is known that miRNAs are a family of naturally occurring small noncoding RNA molecules of 19–24 nucleotides in length that play an important regulatory role in gene expression [1, 2]. They are thought to regulate a large proportion of protein-coding genes [3]. MiRNAs mediate the translational regulation and control gene expression posttranscriptionally by binding to a specific site at the 3′-UTR of target mRNA, which results in mRNA cleavage and translation repression. MiRNAs are transcribed by RNA polymerase II as part of polyadenylated primary transcripts (pri-miRNAs) that can be protein-coding or noncoding. The primary transcripts are then cleaved by the Drosha ribonuclease III enzyme that produce an approximately 70-nucleotide stem-loop precursor miRNA (pre-miRNA), which is further cleaved by the cytoplasmic Dicer ribonuclease (Dcr-1) to generate the mature miRNA and antisense miRNA star (miRNA^*∗*^) products. Further, the mature miRNA is incorporated into RNA-induced silencing complex (RISC), which recognizes the target mRNAs through imperfect base pairing with the miRNA and mostly results in translational inhibition or destabilization of the target mRNA. In general, it has been estimated that about 30% or even more of human mRNAs are regulated by miRNAs [4]. Nearly 2865 mature miRNAs have been identified in the human genome until now, and it is believed that these miRNAs may contribute to at least 60% of the human transcriptome. Recent scientific findings show that miRNAs are emerging as important regulators of cellular differentiation and dedifferentiation and are deeply involved in developmental processes. Moreover, it became evident that dysregulation of miRNA expression may play a fundamental role in progression and dissemination of different cancers. However, it is becoming clear that the activity of miRNAs is not always determined by their expression in cells. Their activity can be affected by RNA-binding proteins such as dead end (DND1), which inhibits the function of variety of miRNAs by blocking the access of target mRNAs [3]. Regarding all this new knowledge, miRNAs may play an important role in modulating gene expression during human preimplantation development from primordial germ cells to the embryo. The interest is still increased by discovery of exosomes, cell-derived vesicles, which are released from the cell when multivesicular bodies fuse with the plasma membrane or are released directly from the plasma membrane [5]. Exosomes can carry different proteins but also miRNAs and mRNAs [6] between different cells and are deeply involved in cell-to-cell communication. They can be found in almost all body fluids and also in media of cell cultures [7]. Exosomes have a great potential to be used for prognosis, therapy, and biomarkers of different diseases including infertility.

The analysis of small RNAs can now be performed by diverse techniques including cDNA cloning and sequencing, real-time PCR, microarrays, and RNA-sequencing. The RNA-seq massively improved and enabled the discovery of novel small RNAs, including miRNAs and sensitive quantitative small RNA expression analysis [8].

The aim of this review paper is to summarize the existent knowledge on miRNAs related to female fertility and cancer: from primordial germ cells and ovarian function, stem cells, oocytes, and embryos that lead to the birth of a new human being or else to aggressive cancers as something goes wrong on this long way. The data on humans reinforced some interesting findings in the animal models.

## 2. Primordial Germ Cells and Germ Cell Tumors

### 2.1. Primordial Germ Cells

The human preimplantation development may be supposed to begin with primordial germ cells (PGCs) which surprisingly arise outside the genital ridge region and can first be identified in the human embryo at about 3 weeks in the yolk sac epithelium near the base of developing allantois [9]. They are recognized by their distinctive morphology and alkaline phosphatase activity. The PGC population is expanded by mitosis and migrates by ameboid movement to the connective tissue of the hind gut and from there into the gut mesentery. About 30 days after fertilization, the majority of the PGCs pass into the region of the developing kidneys and then to adjacent gonadal primordial where they, based on chemotaxis, join the cells of the sex and medullary cords. It has been demonstrated that robust PGC migration is regulated by their miRNAs (e.g., miR-430) in the zebrafish model [10]. Throughout the PGC migration and early colonization, it is not possible to discriminate between female and male gonads and we talk about the indifferent gonads. In the human embryo, the PGC colonization is completed during the sixth week of gestation and after that time the female gonad, ovary, started to develop due to the lack of chromosome Y and the gene SRY, namely, and the PGCs start to develop into female gametes.

The germ cell development requires timely transition from PGCs self-renewal to meiotic differentiation, that is associated with several changes in gene expression, including downregulation of stem cell-associated genes, such as* OCT4* and* KIT*, and upregulation of genes related to germ cells and meiosis, such as* VASA*,* STRA8*, and* SYCP3*. It has already been evidenced that stem cell-expressed RNA-binding protein LIN28 is essential during normal germ cell development for PGC specification in mice. Recently, the expression of LIN28 was examined during normal germ cell development in the human fetal ovary, from the PGC stage, through meiosis and to the initiation of follicle formation [11]. It was found that LIN28 transcript levels were the highest when the fetal ovary contained only PGCs and decreased significantly with increasing time of gestation, concordant with the germ cell differentiation. In addition, immunohistochemistry revealed the expression of LIN28 protein to be germ cell-specific at all stages examined. All PGCs expressed LIN28, but at later gestation time the expression of this protein was restricted to a subpopulation of germ cells, which were demonstrated to be primordial and premeiotic germ cells based on immunofluorescent colocalization of proteins LIN28 and OCT4 and absence of meiosis marker SYCP3. Moreover, the expression of the LIN28 target precursor miRNA transcripts Let-7a/f/d and Let-7g was substantiated in the human fetal ovary, and that expression of these transcripts was the highest at the PGC stage, like for LIN28. The spatial and temporal restriction of LIN28 expression and coincident peaks of expression of LIN28 and target pri-miRNAs suggest important roles for this protein in the maintenance of the germline stem cell state and the regulation of miRNA activity in the developing human ovary. Beside this study, there is almost no data on miRNAs in human PGCs in literature and the main findings came from animal studies.

**Figure 1:**
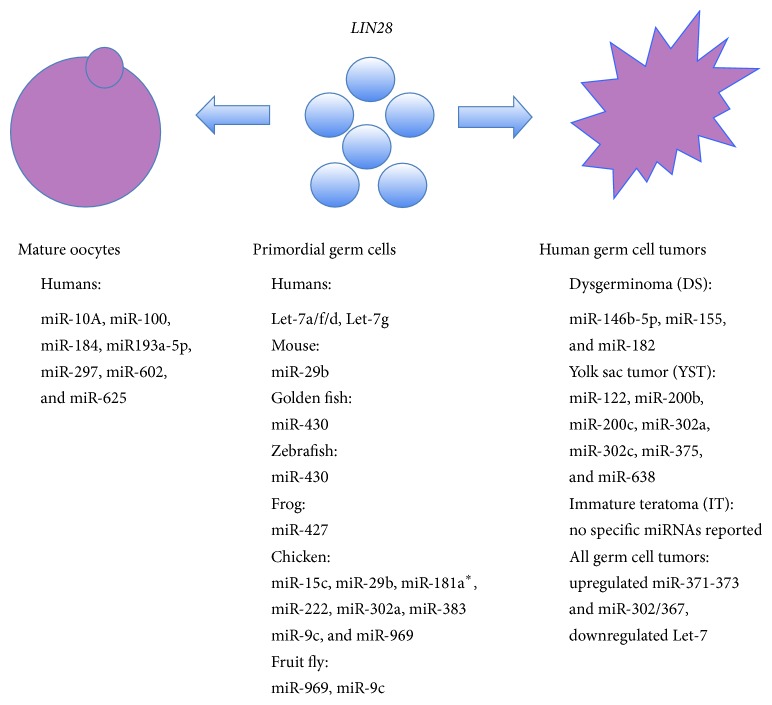
Most expressed miRNAs in primordial germ cells [10–19], germ cell tumors [24], and human oocytes [28, 29]. MiR-29b and miR-430 are overlapping between different vertebrate species. MiR302a, upregulated, and Let-7, downregulated, in germ cell tumors are overlapping with miRNAs identified in vertebrate PGCs. The expression of gene* LIN28* is essential during normal germ cell development for PGC specification. Tumor-suppressor Let-7 miRNA family is downregulated in malignant germ cell tumors because of abundant expression of the regulatory gene* LIN28*.

One of the most important roles of miRNAs in PGCs is targeting the epigenetics-related genes and DNA methylation process in developing gonads. In the mouse model, it has been found that miR-29b may play an important role in female gonadal development by targeting epigenetics-related genes such as* DNMT3A* and* DNMT3B* and thereby modulating methylation of genomic DNA in PGCs [12]. Similarly, it has been found in a chicken, where it has been confirmed that* DNMT3B* expression was reestablished in a female germ cell-specific manner, downregulation by four miRNAs: miR-15c, miR-29b, miR-383, and miR-222 [13]. Some other studies in the vertebrate species such as golden fish [14], zebrafish [15], medaka [16], frog [17], chicken [18], and fruit fly [19] revealed some other miRNAs that may be essential for development and maintenance of PGCs, as can be seen in Figure 1. The pattern of miRNA expression in PGCs seems to be species-specific although some miRNAs such as miR-29b and miR-430 overlap between different species. Some data show that a germline-specific RNA-binding protein DAZ-like (DAZL) acts as an “anti-miRNA factor” during vertebrate germ cell development [15]. During zebrafish embryogenesis, miR-430 contributes to suppress NANOS1 and TDRD7 to primordial germ cells (PGCs) by mRNA deadenylation, mRNA degradation, and translational repression of NANOS1 and TDRD7 mRNAs in somatic cells. It was shown that DAZL can decrease the miR-430-mediated repression of TDRD7 mRNA by inducing poly(A) tail elongation (polyadenylation). These data indicated that DAZL acts as an “anti-miRNA factor” during vertebrate germ cell development. Interestingly, in the case of fruit flies, it was found that embryos derived from miR-969- and miR-9c-mutant mothers had reduced germ cell numbers and increased variance in the phenotype [19] thus indicating that miRNAs may be related to (in)fertility. In addition, it has been confirmed that Dicer1 (Dcr-1) and miRNAs are involved in maintenance and self-renewal of ovarian germinal stem cells in fruit fly ovaries [20–23].

### 2.2. Germ Cell Tumors

PGCs do not always further develop into female gametes but may form uncommon but aggressive and malignant germ cell tumors, which are mostly found in young women or adolescent girls and manifest as different types of malignancies: dysgerminoma (DS), yolk sac tumor (YST), and immature teratoma (IT) [24]. The origin of germ cell tumors traces back to PGCs in the embryo and reflects their specific characteristics such as totipotency and sensitivity to DNA damaging agents; therefore, germ cell tumors provide a useful model to study the gene regulation involved in oncogenesis [25]. Germ cell tumors show some similarities with pluripotent precursor PGCs and stem cells in terms of expression of genes/proteins related to pluripotency (e.g., NANOG, POU5F1, and SOX-2) and embryogenesis (e.g., GATA4, GATA6, and TFAP2C). The molecular characteristics are comparable to DS and testicular counterparts (KIT signaling pathway), while they are quite specific for YST (WNT/B-catenin and TGF-B/bone morphogenetic protein signaling pathways) [24].

The recent data show that dysregulation of miRNAs may be involved in the manifestation of germ cell tumors [24, 26, 27]. Different types of germ cell tumors may develop by different developmental processes involving insufficient sexual differentiation of PGCs and different irregular patterns of miRNA expression, as can be seen in Figure 1. Genomic and protein-coding transcriptomic data have suggested that germ cell tumors (GCTs) in childhood are biologically distinct from those of adulthood. It has been shown that all malignant germ cell tumors overexpress the miR-371–373 and miR-302/367 clusters with increased serum levels, regardless of patient age, histological subtype, or anatomical site of tumor [26, 27]. The tumor-suppressor Let-7 miRNA family has also been shown to be universally downregulated in malignant germ cell tumors because of abundant expression of the regulatory gene* LIN28* and results in upregulation of oncogenes including* MYCN*,* AURKB,* and* LIN28* by itself.

Interestingly, some miRNAs such as miR-29b and miR-430 overlapped between different vertebrate species and some miRNAs upregulated (miR302a) or downregulated (Let-7) in germ cell tumors overlapped with miRNAs identified in vertebrate PGCs according to different studies, as can be seen in Figure 1. MiRNAs identified in PGCs and germ cell tumors differ from miRNAs that were found to be abundant in mature human oocytes [28, 29].

## 3. Ovary: Oocytes, Cumulus (Granulosa) Cells, and Follicular Fluid

### 3.1. Ovary

After migration and colonization, PGCs become oogonia in the forming fetal ovary. The oogonia proliferate extensively by mitotic divisions to up to 5–7 million cells in humans. Each oogonium inside the fetal ovary divides and enters into the initial stage of meiosis to become the primary oocyte. The diploid primary oocyte stopped at the first meiotic prophase stage. It has a nucleus called the germinal vesicle (GV); therefore, this stage refers to the GV-stage of maturity. GV oocytes are localized within the primordial follicles, where they are surrounded by flattened and condensed layer of surrounding granulosa cells. The data derived from the mouse model show that miRNA-376a regulates the primordial follicle assembly in the ovary by modulating the expression of proliferating cell nuclear antigen (*PCNA*) gene in mouse fetal and neonatal ovaries. MiR-376a negatively correlated with PCNA mRNA expression in fetal and neonatal mouse ovaries and directly bound to PCNA untranslated region [30], while there is no data available for humans at present.

By the end of the fetal period, all primary oocytes are formed and stopped at the first meiotic prophase stage. The primary oocytes are maintained for years, until puberty (menarche), when they finish the first meiotic division and divide into two daughter cells: a haploid secondary oocyte and an extruded polar body. During the reproductive period of life, in each menstrual cycle, only a few primary oocytes are recruited, and only one of them indeed matures and is ovulated to be fertilized. When the secondary oocyte enters the second meiotic division, it is not finished but arrested again and held at the metaphase II (MII) stage until fertilization. When the oocyte is fertilized, the process of meiosis is terminated and the second polar body is extruded. The MII-stage oocyte has the potential to be fertilized, while the immature GV-stage oocyte does not. During each menstrual cycle, only 15–20 early antral follicles/oocytes are recruited and only one dominant follicle, indeed, matures and is ovulated. The miRNAs are confirmed to be involved in follicular maturation. Three different miRNAs, miR-224, miR-378, and miR-383, have been found to be involved in the regulation of aromatase expression during follicle development. In addition, miR-21 proved to promote the follicular cell survival during ovulation. Proangiogenic miR-17-5p and let-7b were shown to be essential for normal development of the corpus luteum after ovulation [31]. The experimental data in a mouse model showed that ribonuclease III named Dicer1, essential for synthesis of mature functional miRNAs, is involved in the process of folliculogenesis [32]. Dicer1 protein was expressed in both oocytes and granulosa cells of follicles. The role of miRNAs in mouse ovarian development was explored by using Dicer1 conditional knockout (cKO) mouse in which Dicer1 was deleted specifically in granulosa cells. With Dicer1 deletion, miR-503, that is more abundant in a mouse ovary than in other tissues, was significantly downregulated. The increased pool of primordial follicles accelerated the early follicle recruitment and more degenerated follicles could be observed. The ovarian niche is extremely important for the growth and maturation of follicles/oocytes and irregularities may lead to infertility.

### 3.2. Oocyte-Cumulus Complex

#### 3.2.1. Human Oocytes

**Figure 2:**
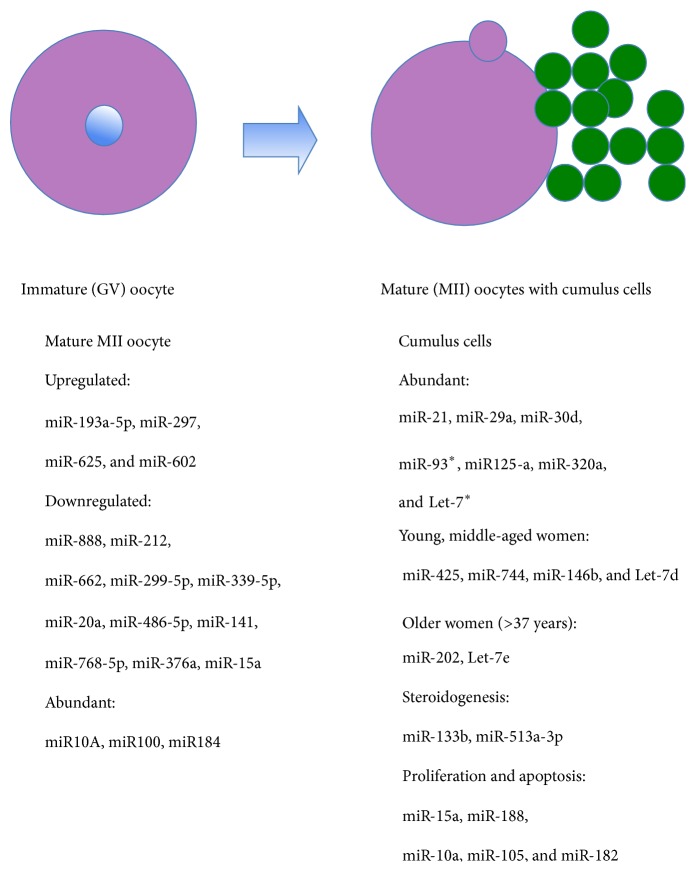
Comparison of miRNAs in immature and mature human oocytes and surrounding cumulus cells according to female age, steroidogenesis, proliferation, and apoptosis [28, 29, 33, 40, 41, 45–47]. Legend: ^*∗*^miRNAs which are also abundant in the mouse cumulus cells.

Each oocyte develops and matures in the functional unit of the ovary, the follicle, surrounded and supported by granulosa and theca cells, which represent the important niche for oocyte growth and maturation. The communication between the oocyte and surrounding follicular cells is a prerequisite for normal growth and maturation of the oocyte resulting in fertilization and the development of an embryo. This oocyte-cumulus communication is still poorly understood and it is proposed that miRNAs may play an important role. There is little data on miRNAs in human oocytes because they do represent a scarce and sensitive biological material, which is mostly not available to researchers. Despite that, miRNAs have already been confirmed in human oocytes retrieved in the in vitro fertilization program in rare studies. In one study, the dynamic changes of miRNAs from GV- to MII-stages were analyzed [28] by miR-CURY LNA microarray platform and quantitative RT-PCR. Oocytes were divided into four groups, corresponding to GV oocytes, MI oocytes, MII oocytes in vitro matured at conventional FSH level (5.5 ng/mL), and MII oocytes matured in vitro at a high FSH level, 2,000 ng/mL, respectively. Altogether, 722 miRNAs were identified in oocytes. In mature MII-stage oocytes, four miRNAs were upregulated and eleven were downregulated in comparison to immature GV-stage oocytes, as can be seen in Figure 2. The RT-PCR analysis of miR-15a and miR-20a expression revealed the concordant dynamic changes of these two miRNAs during meiosis. Moreover, the high concentration of FSH in the in vitro maturation medium led to reverse effect on the expression of miR-15a and miR-20a, which confirmed the involvement of these two miRNAs in the oocyte maturation process influenced by FSH [28]. Another study illustrated that miR10A, miR100, and miR184 were abundant in human mature MII-stage oocytes and that they differed from the palette of miRNAs abundant in surrounding* cumulus oophorus*, a cluster of cells called cumulus cells surrounding the oocyte in the ovarian follicle (see Figure 2) [29].

The predicted target genes of the oocyte miRNAs were associated with the regulation of transcription and cell cycle, while genes targeted by cumulus cell miRNAs were involved in extracellular matrix and apoptosis. The comparison of predicted miRNA target genes and mRNA microarray data resulted in a list of several target genes that were differentially expressed in MII oocytes and cumulus cells, including* PTGS2*,* CTGF,* and* BMPR1B* that are important for cumulus-oocyte communication. Further functional analysis in primary granulosa cell cultures revealed that BCL2 and CYP19A1 mRNA levels were decreased when miR23a is overexpressed. These results suggested that miRNA could play an important role in the regulation of oocyte and cumulus cell cross talk [29]. Interestingly, some of miRNAs that were abundant in human cumulus cells such as miR-93 and Let-7 overlapped with those in mice [33].

Mammalian oocyte maturation is characterized by asymmetric cell division that results in a large oocyte and a small polar body in contrast to symmetric division in mitosis of somatic cells. The asymmetry results from oocyte polarization including spindle positioning, migration, and cortical reorganization, critical for fertilization and for early embryo development. The actin dynamics is involved in this process, and miRNAs are proposed to play an important regulatory role [34].

#### 3.2.2. Animal Oocytes

Because human oocytes are a rare and difficult available research material, some confirmation and interesting findings came from animal experiments in other mammalian species, especially in mouse and bovine (see Supplementary Table 1 in Supplementary Material available online at http://dx.doi.org/10.1155/2016/3984937). Similar to human oocytes, 59 miRNAs were differently expressed in immature and mature bovine oocytes [35]. Moreover, it was found that 47 and 51 of miRNAs were highly abundant in bovine GV and MII oocytes in comparison to the surrounding cumulus cells. Six miRNAs that were enriched in oocytes, namely, miR-96, miR-122, miR-146a, miR-146b-5p, miR-150, and miR-205, dramatically decreased during in vitro maturation [36]. Experiments with the bovine model showed that the expression of an oocyte-specific basic helix-loop-helix transcription factor FIGLA, essential for primordial follicle formation and expression of many genes required for fertilization and early embryonic survival, is regulated by miR-212 [37]. MiR-212 was expressed in oocytes and increased in early embryos. Using transient transfection and reporter assays, it turned out that miR-212 repressed the expression of FIGLA in bovine oocytes and embryos. Both LIN28a and LIN28b transcripts were abundant in mouse oocytes. In marked contrast to vertebrate PGCs, LIN28a and LIN28b were inactive during oocyte growth because their knockdown has no effect on Let-7a levels in intact germinal vesicle (GV) oocytes [38]. Furthermore, transgenic females were fertile and produced healthy offspring, and their overall breeding performance was comparable to that of wild-type mice. In spite of several miRNAs identified in mouse oocytes, some experimental data revealed that miRNA function may be globally suppressed in mouse oocytes and early embryos and that another type of small RNAs, small interfering RNAs (siRNAs), may play an important role [39]. It is known that the deletion of* Dgcr8, *also known as* Pasha* that is required for miRNA processing (binds to Drosha), specifically blocks the production of canonical miRNAs, while the deletion of* Dicer *blocks the production of both miRNAs and endo-siRNAs in cells. The conditional deletion of* Dicer *in mouse oocytes leads to severe defects in chromosomal alignment, spindle organization, and infertility. In contrast to* Dicer*,* Dgcr8*-deficient mouse oocytes matured normally and produced healthy offspring after fertilization. Their mRNA profiles were identical to oocytes of wild-type mice, while* Dicer-*null oocytes showed several misregulated transcripts. This phenomenon needs to be further elucidated in humans and other mammalian species.

### 3.3. Cumulus and Granulosa Cells

The data on human cumulus cells with predominating granulosa cells are much more abundant, because it is easier to retrieve the cumulus cells in the in vitro fertilization program than oocytes by themselves. The cumulus cells can be obtained mechanically (cutting) or by enzymatic (hyaluronidase) denudation of oocytes and are otherwise discarded in daily medical practice. Cumulus cells can be studied from various angles as they are related to oocyte maturation and quality. A large-scale profiling of EIF2C2- (protein argonaute-2-) bound miRNAs was performed in three human granulosa-derived cell lines (i.e., KGN, HSOGT, and GC1a) and in primary human granulosa cells using high-throughput sequencing [40]. Argonaute proteins with their argonaute-2 containing PIWI domain have a dominant effect on RNA interference. They interact with Dicer1 and participate in short-interfering-RNA-mediated gene silencing. MiR-21 accounted for more than 80% of EIF2C2-bound miRNAs thus suggesting that it was enriched in the RNA-induced silencing complex (RISC) and was important for human granulosa cells. The target of miR-21 in granulosa cells was COL4A1 mRNA that is related to a component of the basement membrane surrounding the granulosa cell layer and the granulosa-embedded extracellular structure.

#### 3.3.1. Female Age

The increase in female age is the main factor reducing female fertility and the outcome of assisted conception. The mechanisms are still not well understood. It has been found that female aging alters the expression of variety of genes in human cumulus cells being essential for oocyte quality and potential targets of specific miRNAs previously identified in cumulus cells, such as miR-425, miR-744, miR-146b, and Let-7d for younger (<30 years) and middle-aged (31–34 years) women and miR-202 and Let-7e for elder (>37 years) women [41].

#### 3.3.2. Follicle and Steroidogenesis

Some studies demonstrated that miRNA pattern is a dynamic feature in a follicle and may differ between different groups of follicular cells surrounding and supporting the oocyte. In more detail, there was a difference in the miRNA pattern between the* corona radiata*, the innermost layer of the* cumulus oophorus,* directly adjacent to the oocyte zona pellucida, and the rest of the cumulus cells [42]. A total of 785 and 799 miRNAs were identified in* corona radiata* and the rest of the cumulus cells. Seventy-two miRNAs were differently expressed between these two groups of cells. The bioinformatics analysis showed that these miRNAs were related to amino acid and energy metabolism. Another study determined the miRNA profile of two intrafollicular somatic cell types: mural (MGCs from the outer “wall” of the follicle) and cumulus (CGCs inner cells around the oocyte) granulosa cells from women undergoing controlled ovarian stimulation for in vitro fertilization [43]. Altogether, 936 annotated miRNAs and nine novel miRNAs were identified, and 90 of the annotated miRNAs were differentially expressed between MGCs and CGCs. The bioinformatic prediction revealed that different pathways, such as TGF*β*, ErbB signaling, and heparan-sulfate biosynthesis, were targeted by miRNAs in both granulosa cell populations, while extracellular matrix remodeling, Wnt, and neurotrophin signaling pathways were enriched among miRNA targets in MGCs. Interestingly, two of the nine novel miRNAs identified were found to be of intronic origin: one from the aromatase and the other one from the FSH receptor gene; the latter miRNA was predicted to target the activin signaling pathway. These results suggest that posttranscriptional regulation of gene expression by miRNAs may play an important role in the modification of gonadotropin signaling [43]. This is in accordance with the general knowledge on granulosa cells. Within the follicle, the granulosa cells surrounding the oocyte are in intimate contact with it by gap and adhesion junctions and release several substances decisive for oocyte growth and maturation. Granulosa cells are also involved in the endocrine activity of follicle, steroidogenesis, by transforming the androgens (androstenedione) from theca cells into estrogens by aromatase activity. They possess FSH hormone receptors and the number of granulosa cells increases directly in response to the increased levels of circulating gonadotropins or decreases in response to testosterone. They also produce some peptides involved in the regulation of ovarian hormone synthesis. Furthermore, the effect of transfection of cultured primary human ovarian granulosa cells with 80 different gene constructs encoding human pre-miRNAs on release of hormones, progesterone, testosterone, and estradiol, was evaluated by enzyme immunoassay [44]. In addition, two selected antisense constructs, blocking the corresponding miRNAs, were tested on progesterone release in granulosa cells. It has been shown that 36 out of 80 tested miRNA constructs resulted in inhibition of progesterone release and 10 miRNAs promoted progesterone release in human granulose cells. Fifty-seven miRNAs tested inhibited testosterone release, and only one miRNA enhanced testosterone output and 51 miRNAs suppressed estradiol release, while none of the miRNAs tested stimulated it. The authors suggested that miRNAs can control reproductive functions by enhanced or inhibited release of ovarian progestagen, androgen, and estrogen in human granulosa cells and suggested that such miRNA-mediated effects could be potentially used for the regulation of reproductive processes including fertility and for the treatment of reproductive and other steroid-dependent disorders of women [44]. The proof was given that miR-133b downregulates* FOXL2* expression by directly targeting the 3′UTR and inhibiting the FOXL2-mediated transcriptional repression of* StAR* and* CYP19A1* to promote estradiol production in the mouse granulosa cells [45].

It has also been found that miR-513a-3p is involved in the control of the luteinizing hormone/chorionic gonadotropin receptor (LHCGR) expression by an inversely regulated mechanism at the posttranscriptional level, essential for normal female reproductive function [46].

#### 3.3.3. Proliferation and Apoptosis

One of the most important factors affecting the oocyte quality is apoptosis of granulosa cells surrounding the oocyte. The granulosa cell apoptosis can be related to impaired oocyte quality. To better elucidate the potential role of miRNAs in the regulation of proliferation and apoptosis in ovarian granulosa cells, the effect of transfection of cultured primary human granulosa cells with 80 different constructs encoding human pre-miRNAs on the expression of the proliferation marker, PCNA, and the apoptosis marker Bax was evaluated using immunocytochemistry [47]. The results showed that eleven out of 80 tested miRNA constructs resulted in stimulation, and 53 miRNAs resulted in inhibition of PCNA. Moreover, 11 out of the 80 miRNAs tested promoted accumulation of Bax, while 46 miRNAs caused a reduction in Bax in human granulosa cells. In the next step, two selected antisense constructs blocking the corresponding miRNAs miR-15a and miR-188 were used to evaluate their effects on the expression of PCNA. An antisense construct inhibiting miR-15a increased PCNA, while an antisense construct of miR-188 did not affect PCNA expression. Validation of effects of selected pre-miR-10a, miR-105, and miR-182 by using other markers of proliferation (cyclin B1) and apoptosis (TdT and caspase 3) confirmed the specific role of miRNAs in human granulosa cell proliferation and apoptosis [47].

### 3.4. Follicular Fluid and Plasma

It is of great advantage that miRNAs can be identified in body fluids such as follicular fluid retrieved in the in vitro fertilization program or in blood plasma. Follicular fluid is retrieved by ultrasound-guided aspiration of oocytes from follicles after controlled hormonal stimulation of ovaries for in vitro fertilization. When the oocytes are removed for in vitro fertilization, the follicular fluid is discarded in daily medical practice but can represent a perfect material to study the ovarian physiology and pathologies and the oocyte quality. It has been reported that miRNAs can readily be detected within membrane-enclosed vesicles of human follicular fluid. In a current research, 37 miRNAs were found to be upregulated in human follicular fluid in comparison to plasma in the same women [48]. It was evidenced that 32 of these miRNAs were carried by microvesicles, exosomes, that expressed the well-characterized exosomal markers such as CD63 and CD81. The authors suggested that these miRNAs identified in the follicular fluid are involved in the critically important pathways for follicle growth and oocyte maturation. Specifically, nine of these miRNAs are known to target and negatively regulate mRNAs expressed in the follicular microenvironment by encoding inhibitors of follicle maturation and meiosis resumption. It has been evidenced that increased female age affected the pattern of miRNAs in follicular fluid [49]. In the miRNA profile of the follicular fluid of younger (<31 years) and older (>38 years) women, there was a set of four differentially expressed miRNAs. The predicted targets of these miRNAs were genes involved in the heparan-sulfate biosynthesis, extracellular matrix-receptor interaction, carbohydrate digestion and absorption, p53 signaling, and cytokine-cytokine-receptor interaction. It needs to be exposed that several of these pathways are related to fertility, suggesting that this set of miRNAs and their targets need to be studied in relation to reproductive aging and assisted conception outcome.

Moreover, it has been found that ovarian pathologies such as polycystic ovary syndrome (PCOS) and premature ovarian failure (POF) or primary ovarian insufficiency affect the miRNA expression in follicular fluid and plasma.

#### 3.4.1. Polycystic Ovary Syndrome

**Figure 3:**
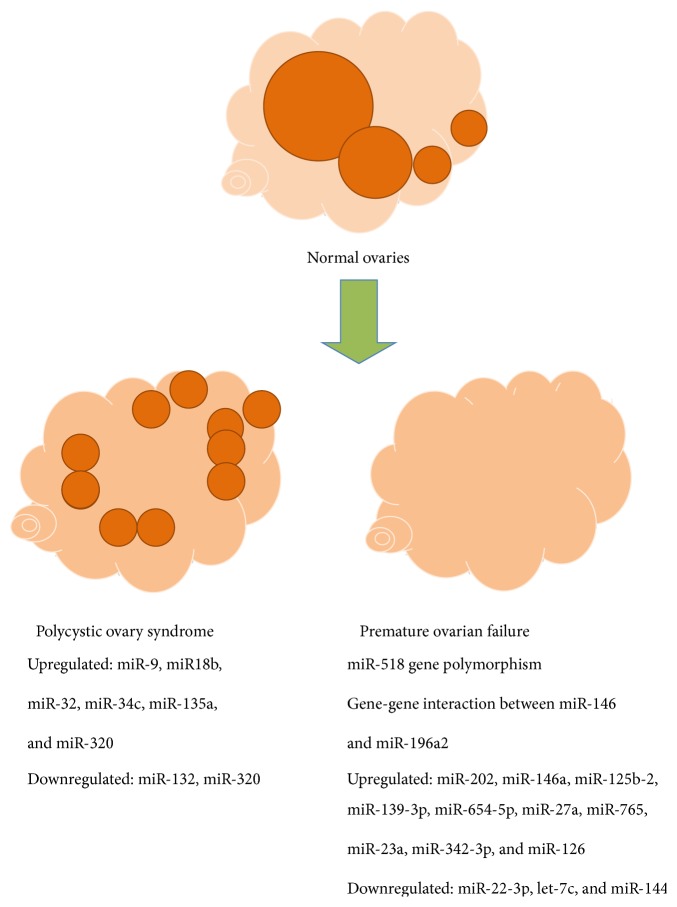
MiRNAs that were differently expressed in follicular fluid and plasma of women with polycystic ovary syndrome [51–54] and premature ovarian failure [55–60] in comparison to healthy women with normal ovaries.

PCOS is a systematic disease represented by a set of symptoms due to hormone imbalance in women [50]. It is usually diagnosed by anovulation, high androgen levels, and several ovarian cysts detected by ultrasound. The typical symptoms include irregular or no menstrual periods, heavy periods, obesity, excess body and facial hair (hirsutism), acne, pelvic pain, low fertility and trouble getting pregnant, and patches of thick and darker skin. It can be related to the type 2 diabetes, obstructive sleep apnea, heart disease, mood disorders, and endometrial cancer. Moreover, PCOS is one of the most common endocrine disorders in women of reproductive age and causes low fertility. Microarray profiling of human follicular fluid revealed the expression of 235 miRNAs in human follicular fluid and 29 of them were differentially expressed between the PCOS and normal groups of women [51]. After PCR validation, 5 miRNAs showed significantly increased expression in the PCOS group of women (Figure 3). The potential target genes were related to insulin regulation and inflammation. Moreover, the most highly expressed miRNA targeted genes were found to be associated with reproductive, endocrine, and metabolic processes. In another study, it has been found that two miRNAs, miR-132 and miR-320, were expressed at significantly lower levels in the follicular fluid of women with PCOS than in a group of healthy women, as can be seen in Figure 3. In addition, it has been evidenced that miR-132, miR-320, miR-520c-3p, miR-24, and miR-222 that are present in the follicular fluid regulate estradiol concentrations and miR-24, miR-193b, and miR-483-5p progesterone concentrations [52]. The authors concluded that there are several miRNAs in human follicular fluid and that some of them may play an important role in steroidogenesis and PCOS. Interestingly, the miR-320 was differently expressed in the follicular fluid of women with PCOS in still one study [53] and was along miR-383 upregulated in comparison with healthy (control) women. This study demonstrated that miR-320 regulates the proliferation and steroid production by targeting E2F1 and SF-1 in granulosa cells and is involved in the follicular development. The authors summarized that understanding the regulation of miRNA biogenesis and function in the follicular development in humans will potentiate the usefulness of miRNA in the treatment of some steroid-related disorders and female infertility. In comparison to healthy women, a total of 59 known miRNA were identified that were differentially expressed in cumulus granulosa cells of women with PCOS: 21 miRNAs were increased and 38 miRNAs decreased [54]. Several important processes could be targeted by these miRNAs, such as Notch signaling, regulation of hormones, and energy metabolism. Moreover, Notch3 and MAPK3, the members of Notch signaling and ERK-MAPK pathway, were found to be directly regulated by miR-483-5p.

#### 3.4.2. Premature Ovarian Failure

Another indication of severe ovarian infertility is POF, which is defined as an ovarian disorder of multifactorial origin characterized by amenorrhea, hypergonadotropism (e.g., increased FSH levels), hypoestrogenism, and no follicles/oocytes to be fertilized in women under the age of 40 years [55]. The plasma samples are usually analyzed on miRNAs in patients with POF because they mostly do not have follicles. In one study, the microarray chip results demonstrated that 10 miRNAs were significantly upregulated and 2 miRNAs were downregulated in plasma of POF patients compared with normal women, as can be seen in Figure 3 [56]. Among miRNAs that were upregulated in plasma was miR23a, which has been confirmed to be of great importance in regulation of apoptosis in ovarian granulosa cells via decreasing X-linked inhibitor of apoptosis protein (XIAP) expression [57]. Further, miR-22-3p has been found to show a lower expression level in the plasma of women with POF and distinguish them from control subjects. In addition, it was negatively associated with serum FSH levels [58]. The target functions of miR-22-3p were apoptosis, endocytosis, and tumorigenesis. The authors suggested that decreased expression of miR-22-3p in plasma of POF patients reflects the diminished ovarian reserve as a consequence of the pathologic process of POF. Further, the current study provided the evidence to implicate miR-518 and TGFBR2 gene polymorphisms as novel susceptibility factors for POF, age at natural menopause, and early menopause risk [59]. Some findings suggest that putative gene-gene interaction between miR-146 and miR-196a2 may be involved in POF development [60]. It is very important to study the causes of diminished ovarian reserve. Actually, there are no relevant biomarkers available to estimate the ovarian reserve in (in)fertile women and to predict the assisted conception outcome. The existing ultrasound monitoring of ovaries and the determination of FSH and AMH hormone levels in serum have been proven to be insufficient and nonreliable. Therefore, some new biomarkers are needed.

## 4. Fertilization and Paternal Contribution to the Embryo

Fertilization is the fusion of gametes to initiate the development of a new individual organism and represent one of the main events in the human preimplantation development. Naturally, it occurs in the ampullary region of the oviduct but may also occur in vitro. During fertilization, sperm contribute some genetic and epigenetic factors that affect the early embryogenesis [61]. Epigenetic factors contributed by sperm include a functional centrosome, proper packaging of the chromatin with sperm-specific protamines, modifications of histones, and imprinting of genes. In addition, the fertilizing spermatozoon provides its own mRNAs and miRNAs, which may contribute to the embryonic transcriptome and regulate the embryonic gene expression. All these epigenetic factors may directly or indirectly affect the expression of genes in the developing embryo.

In general, spermatozoa are transcriptionally inactive, extremely specialized haploid cells with a head containing a compact nucleus and minimal cytoplasm. On the other hand, especially during recent years, it has been proven that sperm still contain a complex population of RNAs that comprises rRNA, mRNA, and both long and small noncoding RNAs. It was suggested that the intact mRNA sequences are enriched for genes associated with the infertility, fertilization, and early embryo development [8]. It has been demonstrated that mRNA is retained in sperm and increases after fertilization prior to zygotic genomic activation [62]. rRNA is the most enriched RNA population but highly fragmented thus ensuring the translationary inactivated state of the sperm cell.

Gradually, the overall functional significance of RNA in sperm is better recognized. Using the zona pellucida free hamster oocyte/human sperm penetration assay, it has been shown that sperm-specific transcripts, not present in the unfertilized oocyte, are transmitted to the oocyte upon fertilization [63]. Further, it has been demonstrated that injection of a sperm-borne transcript PLC-zeta, not present in the unfertilized oocyte, can be translated in the oocyte into a functional calcium oscillator and oocyte activator during fertilization [64]. The presence of noncoding RNAs in sperm also postulated potential roles in early postfertilization and embryonic development. These assumptions have been extended to the view that sperm-borne RNAs might have epigenetic effects on the phenotype of the developing organism. The region of mature sperm chromatin that harbors imprinted genes exhibits a dual nucleoprotamine/nucleohistone structure with DNase-sensitive regions that might be implicated in the establishment of efficient epigenetic information in the developing embryo [65, 66].

**Figure 4:**
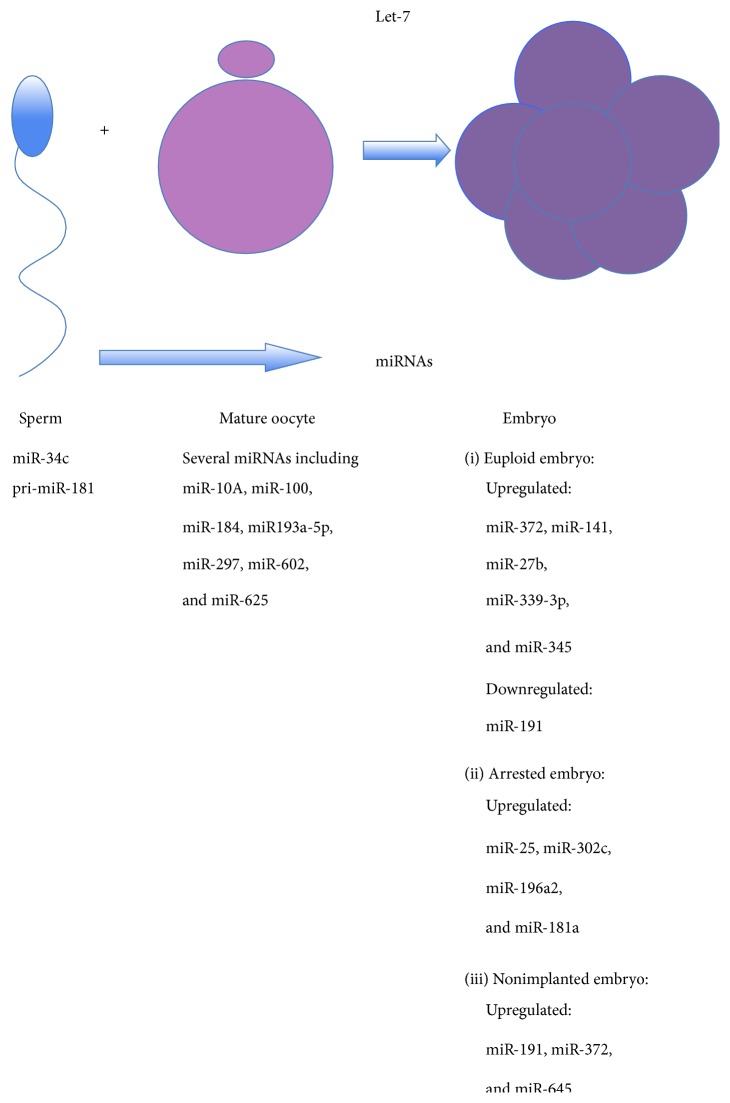
MiRNAs in human sperm, mature oocytes, and embryos, important for fertilization and embryo development [75–80]. Some miRNAs identified in sperm are contributed to the oocyte during the fertilization process and are involved in embryogenesis. Abundant maternal miRNAs of Let-7 family are involved in oocyte-to-embryo transition and are replaced by embryonic miRNAs, related to pluripotency.

MiRNAs are the most characterized noncoding sperm RNAs. It has been suggested that miRNAs in sperm (Figure 4) might function in embryonic histone replacement [67] or transcriptionally balance the genome for early expression signaling or effect epigenetic modification [68] since nearly 10% of all small noncoding RNAs map to histone enriched TSS and promoter regions. MiR-34c is a highly abundant miRNA in humans [69]. It has been revealed that it is essential for early embryo development and required for the first cleavage division of the zygote in mice. Also miRNA precursors are present in human sperm, such as pri-miR-181, whose targets might have a function in early embryonic development and globally decrease at the 4–8-cell stage of human embryo development [8]. A specific target of pri-miRNA-181 is the embryonic stem cell pluripotency factor, termed coactivator-associated arginine methyltransferase I (CARM1). With the mouse model, it was demonstrated that miRNAs are present in mouse sperm structures that enter the oocyte at fertilization. The sperm contained a broad profile of miRNAs and a subset of potential mRNA targets, which were expressed in fertilizable metaphase II (MII) oocytes [70]. However, the levels of sperm-borne miRNAs were low in comparison to those of unfertilized MII oocytes, and fertilization did not alter the oocyte miRNA repertoire that included the most abundant sperm-borne miRNAs. In general, after fertilization, potential mechanisms and functions of sperm RNA could include translation of intact paternal mRNAs by the MII oocyte machinery, translational regulation by paternal miRNAs, activation of paternal pri-miRNAs by maternal Dicer, and transcriptional regulation by paternal miRNAs. Noncoding RNAs have been postulated as epigenetic modifiers including histone modification and DNA methylation and playing a function in transgenerational epigenetic inheritance. The profiling of small RNAs by highly sensitive techniques, such as next generation sequencing and real-time PCR, might have some potential for the discovery of new fertility markers in human medicine [71]. The comparison of differential expressed small RNAs might lead to the discovery of new regulatory pathways underlying male infertility. The possibility that the profile of small noncoding RNA in sperm could be controlled by environmental factors, such as paternal stress exposure [72], early trauma [73], and pollution [74], also shed new light and speculations on the determination of epigenetic traits to the next generation.

## 5. Embryo

The oocyte-to-embryo transition means the transformation of a highly differentiated oocyte into a totipotent zygote and pluripotent blastomeres of the preimplantation mammalian embryo. It includes the replacement of abundant maternal miRNAs that are enriched also in differentiated cells and exemplified by the Let-7 family, with embryonic miRNAs that are common in pluripotent stem cells (e.g., miR-290 family in the mouse) [38], as can be seen in Figure 4. The fertilization process is followed by the development of an embryo mainly developed on the basis of maternal mRNAs. The embryo starts to express its own genome at the stage of 4–8 cells before implantation. It develops through the morula stage with approximately 30 cells to the blastocyst stage with approximately 100 cells. The human embryo usually implants at the late morula or blastocyst stage. The role of miRNAs in early mammalian development, and particularly in the posttranscriptional regulation of maternal mRNAs, is still controversial. With the mouse model, it has been illustrated that the capacity to process the miRNAs diminishes after fertilization thus reducing the miRNA activity in the later stages of preimplantation development [75]. However, by analyzing the different precursor and mature forms of specific miRNAs that are abundant in the mouse blastocyst, such as miR-292-3p and miR-292-5p, it has been found that miRNA-duplexes and/or miRNAs bound to target mRNAs can appear and may serve as potential stock of miRNAs in the developing embryo. It has been suggested that such stockpile could directly provide functional and mature miRNAs in response to demand [75]. This phenomenon needs further research.

### 5.1. Genetic Status of Embryos

Human embryos have already been analyzed on miRNAs and it has been demonstrated that the miRNAs expression pattern depends on the chromosomal status (Figure 4). In one study, it has been found that the miRNA patterns of euploid embryos, normal for all chromosomes, differ from aneuploid embryos in humans [76]. The most highly expressed miRNA in euploid embryos was miR-372, as revealed by an array-based quantitative real-time polymerase chain reaction (qPCR). Several of the highly expressed miRNAs have shown to be critical to the maintenance of stem cell pluripotency and mammalian embryo development. There were some miRNAs, differently expressed between euploid and aneuploid embryos, such as miR-141, miR-27b, miR-339-3p, and miR-345 that were all upregulated in euploid embryos.

### 5.2. Embryo Development and Implantation

Surprisingly, it has been demonstrated that human embryos secrete miRNAs into culture media and that miRNAs may represent new biomarkers for embryo development and implantation [77, 78]. In human and bovine, differential miRNA gene expression was observed in (spent) media after culture of embryos that developed to the blastocyst stage and those that were arrested and failed to develop from the morula to the blastocyst stage. In spent media, miR-25, miR-302c, miR-196a2, and miR-181a expression were found to be higher in arrested embryos than in blastocysts [77], as can be seen in Figure 4.

The spent culture media from 55 single-embryo transfer cycles were tested for miRNA expression using an array-based quantitative real-time polymerase chain reaction analysis and the expression of the identified miRNA was correlated with pregnancy outcomes in these cycles [78]. Two miRNAs, miR-191 and miR-372, were expressed specifically in spent media after embryo culture. Interestingly, miRNAs were related to some bad condition: miR-191 was more highly concentrated in media from aneuploid embryos, and miR-191, miR-372, and miR-645 were more highly concentrated in media from failed in vitro fertilization cycles without pregnancy (Figure 4). In addition, miRNAs were found to be more highly concentrated in media after intracytoplasmic sperm injection (ICSI) and day-5 media samples when compared to media after classical in vitro fertilization with oocyte insemination (IVF) and day-4 samples, respectively [78]. Embryo implantation also depends on the endometrial receptivity. The repeated implantation failure (RIF) is one of the major problems encountered in the in vitro fertilization program. Thirteen miRNAs have been identified being different in RIF endometrial samples, compared to normal ones, and putatively regulate the expression of 3800 genes [79]. It was found that ten miRNAs were overexpressed in RIF endometrial samples, including miR-23b, miR-99a, and miR-145, whereas three were underexpressed. According to the miRNA-predicted targets, mRNA levels related to the cell adhesion molecules, Wnt signaling, and cell cycle pathways were lower in RIF-IVF patients. With the mouse model, it has been shown that a minimal uterine expression of miR-181 is essential for the onset of embryo implantation and that it is regulated by the leukemia inhibitory factor (LIF) [80].

### 5.3. Human Embryonic Stem Cells

Human embryonic stem cell (hESC) lines can be created from supernumerary embryos from the in vitro fertilization program. They can be retrieved by isolation and in vitro culture of blastocyst inner cell mass (ICM) which otherwise develops into embryonal tissues after implantation. The creation of hESC lines and their research has answered several important questions about pluripotency and stem cell self-renewal and differentiation. Moreover, several studies show that dysregulation of miRNA expression may play a fundamental role in progression and dissemination of different cancers.

#### 5.3.1. Pluripotency

The miR-302/367 cluster represents the most abundant cluster of eight miRNAs that are specifically expressed in hESCs [81]. Functional studies identified important roles of miR-302/367 in regulation of pluripotency and differentiation of hESCs in vitro. Beside its role in TGF-*β* signaling, miR-302/367 also promotes the bone morphogenetic protein (BMP) signaling. Several studies [82–87] have shown that miRNAs are deeply involved in the balance between pluripotency and differentiation of hESCs by regulating the genes related to pluripotency, as can be seen in Table 1.

#### 5.3.2. Differentiation and Development of hESCs into All Three Germ Layers

Interestingly, there is a cluster of primate-specific miRNAs (ps-miRNAs), a set of 269 ps-miRNAs that are evolutionarily conserved and may contribute to the difference between high-level primates and nonprimate mammals or lower vertebrates [88]. They are enriched in chromosomes 19 and X and represented by the miR-548 family. Most ps-miRNAs were low expressed in adult tissues but were highly expressed in reproductive system and hESCs. The target genes were strongly associated with developmental processes and various cancers. It is suggested that ps-miRNAs may play critical roles in regulating of differentiation and growth during the early development and in maintaining the pluripotency of hESCs.

Several studies [89–93] proved that miRNAs are deeply involved in the differentiation of hESCs into different types of cells of all three germ layers (endoderm, mesoderm, and ectoderm), as can be seen in Table 2.

Some miRNAs are involved in maintaining the pluripotency of hESCs but direct their differentiation into a germ layer when they are upregulated [90].

All these recent findings are extremely important for the understanding of the early human development and the improvement of hESC differentiation protocols.

#### 5.3.3. Cancer

If something goes wrong during the process of development and miRNAs are dysregulated, different types of cancers can occur. Oncogenic miRNAs are involved in manifestation of cancers, while another group of suppressive miRNAs is involved in the suppression of cancers [94]. A new mechanism of telomerase regulation by means of noncoding small RNAs has been revealed. It has been shown that miR-498 induced by vitamin D3 decreases the mRNA expression of human telomerase reverse transcriptase [95]. The levels of miR-498 expression are decreased in malignant human ovarian tumors as well as in human ovarian cancer cell lines.

The protein LIN28 is an evolutionarily conserved RNA-binding protein and is known to be a master regulator controlling the pluripotency of hESCs. Together with OCT4, SOX2, and NANOG, LIN28 can reprogram somatic cells and produce induced pluripotent stem cells (iPSCs). The expression of LIN28 is restricted to ESCs and developing tissues and is highly upregulated in human tumors. It functions as an oncogene promoting malignant transformation and tumor progression. It has been demonstrated that four miRNAs, let-7, miR-9, miR-30, and miR-125, directly repress the LIN28 expression in hESCs and cancer cells [96]. It has been suggested that global downregulation of these miRNAs may be one of the key mechanisms of LIN28 reactivation and manifestation of human cancers.

Dicer1, an endoribonuclease that is essential for the synthesis of miRNAs, also acts as a tumor suppressor. It has been demonstrated that inactivation of its RNase IIIB domain by mutation of D1709, a residue mutated in a subset of nonepithelial ovarian cancers, results in complete loss of 5p-derived mature miRNAs, including the tumor-suppressive let-7 family and the consequent progression of cancer [97].

### 5.4. Female (In)Fertility

Recently, the role of Dicer function in female reproductive tissues has begun to be elucidated by the use of knockout mouse models. As already mentioned, the ribonuclease III endonuclease, Dicer1 (also known as Dicer), is essential for the synthesis of miRNAs. Although it is generally established that miRNAs are expressed in the female reproductive tract, their functional role and effects on reproductive processes remain unknown. In an interesting study, the reproductive phenotype of mice with loxP insertions in the Dicer1 gene (Dicer1fl/fl) when crossed with mice expressing Cre-recombinase driven by the anti-müllerian hormone receptor 2 promoter (Amhr2Cre/+) was established for the first time [98]. Adult female Dicer1fl/fl;Amhr2Cre/+ mice displayed normal mating behavior but did not produce offspring after mating in experimental condition. Morphological and histological assessments of the reproductive tracts of mice showed that their uterus and oviducts were hypotrophic and highly disorganized. Natural mating of Dicer1fl/fl;Amhr2Cre/+ females resulted in successful fertilization but the oviductal transport of embryos was disrupted, as evidenced by the failure of embryos to enter the uterus. These data implicated that Dicer1/miRNAs mediated posttranscriptional gene regulation in reproductive somatic tissues that is critical for female fertility. The loss of Dicer within the oocyte, the ovarian granulosa cells, the luteal tissue, the oviducts, and, potentially, also the uterus causes female infertility [99].

It has been shown that miR-290–295, a mammalian-specific cluster of miRNAs, plays a decisive role in embryonic development, as indicated by the partial lethality of mutant mouse embryos [100]. In surviving miR-290–295-deficient mouse embryos, only female fertility was compromised. It has been suggested that this fertility impairment arises from a defect in migrating PGCs and occurs equally in male and female mutant animals. However, male miR-290–295(–/–) mice were able to recover from this initial germ cell loss due to the extended proliferative lifespan of their germ cells and are fertile, while female miR-290–295(−/–) mice are unable to recover and are sterile because of premature ovarian failure.

## 6. Diagnostics and Treatment of Female Infertility: Prospects

The new knowledge and methodology on miRNAs may provide some new biomarkers of female infertility from different aspects: from ovarian physiology to the oocyte and embryo quality, embryo implantation potential, and endometrial receptivity. An important task remains to study the causes of diminished ovarian reserve because there are no relevant biomarkers available to estimate the ovarian reserve in (in)fertile women and to predict (or explain) the assisted conception outcome. miRNAs seem to be an extremely interesting tool to do that. Some of these miRNAs as well as the exosomes, vesicles transporting them, can be easily detectable in the bloodstream and could be used as reliable biomarkers of interest in infertility care. The data show that miRNAs can control reproductive functions by enhanced or inhibited release of ovarian progestagen, androgen, and estrogen in human granulosa cells and suggest that such miRNA-mediated effects could be potentially used for regulation of reproductive processes and for the treatment of reproductive and other steroid-dependent disorders in women with fertility problems.

At present, the oocytes and embryos retrieved in the in vitro fertilization program can be evaluated only by morphology or preimplantation genetic diagnostics after “aggressive” embryo biopsy. The secretion of miRNAs into the culture medium may represent an interesting noninvasive tool to evaluate the oocytes and embryos in the in vitro fertilization program. Moreover, the studies with animal models show that some suboptimal procedures, such as in vitro maturation of ovarian follicles, could be improved using miRNAs. It has been demonstrated that hCG supplementation and vitamin C status in the culture medium alter the miRNA expression profiles in oocytes and granulosa cells during in vitro growth of murine follicles [101]. Moreover, the transfection of murine granulosa cells with let-7c-, miR-27a-, and miR-322-inhibitor sequences increased the oocyte maturation rate by 1.5- to 2.0-fold in comparison with control during in vitro maturation of mouse follicles [102]. These findings suggest that sophisticated miRNA regulation in granulosa cells might improve oocyte maturation efficiency during ovarian follicle development in vitro. Last but not least, the new knowledge on miRNAs in hESCs leads to better understanding of the human development on the one hand and the manifestation of cancer on the other hand. This may result in optimized in vitro differentiation protocols and treatment of different cancers.

## 7. Conclusion

It may be concluded that miRNAs represent a great challenge in reproductive and regenerative medicine to understand the preimplantation development in humans including female reproductive tissues, PGCs, gametes, embryos, and embryonic stem cells better. The forthcoming new knowledge may provide new biomarkers and treatments of fertility disorders and degenerative diseases in the future.

## Supplementary Material

Supplementary Table 1: References on miRNAs in animal oocytes and their biological functions.

## Figures and Tables

**Table 1:** MiRNAs regulating pluripotency and differentiation of hESCs.

Genes related to pluripotency						References
*OCT4*	*NANOG*	*SOX2*	*KLF4*	*LEFTY*	*NR2F2*	
*Maintenance of pluripotency*						
miR-203	miR-203					[82]

*Regulation of stem cell renewal and differentiation*						
	miR-200c					[83]

*Delay of differentiation, balance between differentiation and pluripotency*						
				miR-302s		[84]

*Low in self-renewal and high during differentiation*						
miR-145		miR-145	miR-145			[85]

*Regulation of cell cycle genes in pluripotent stem cells*						
miR-302a		miR-302a				[86]

*Balance between pluripotency and differentiation, NR2F2 critical for neural gene activation*						
miR-302					miR-302	[87]

**Table 2:** MiRNAs and their target genes related to differentiation of hESCs, development of *all three* germ layers *(endoderm, mesoderm, and ectoderm*)*, and cancer*.

Development						References
Endoderm	Mesoderm	Cardiomyocytes	Neural lineage	Endothelial cells, angiogenesis	CANCER	
miR-302a (*OTX2*)						[89]

	miR-302/373 (*LEFTY*)					[90]

			miR-302 (*NR2F2, JMJD1C*)			[91]

		miR-1, miR-499				[92]

				miR-126, miR-210		[93]

					miR-498	[95]

					let-7, miR-9, miR-30, and miR-125	[96]

					let-7 family	[97]
